# Hygroscopic Behavior of Polypropylene Nanocomposites Filled with Graphene Functionalized by Alkylated Chains

**DOI:** 10.3390/nano12234130

**Published:** 2022-11-23

**Authors:** Dongwoo Kang, Sung Hee Kim, Donghyeok Shin, Ji Taek Oh, Myeong-Gi Kim, Pyoung-Chan Lee

**Affiliations:** 1 Chemical Materials R&D Department, Korea Automotive Technology Institute, Cheonan-si 31214, Republic of Korea; 2 School of Chemical Engineering, Sungkyunkwan University, Suwon-si 16419, Republic of Korea; 3 R&D Center, Woosung Chemical Co., Ltd., Cheonan-si 31214, Republic of Korea; 4 R&D Center, BESTGRAPHENE Co., Ltd., Yeoju-si 12616, Republic of Korea

**Keywords:** nanocomposites, graphene, moisture, automotive

## Abstract

Owing to stringent international environmental and fuel efficiency requirements for lightweight automotive systems, polymer composites have attracted widespread attention. Polypropylene (PP) is a widely employed commercial polymer because of its lightweight and low cost. In this study, PP nanocomposites were fabricated to reduce the moisture absorption of PP composites in automotive headlamp housings. Alkylated chemically modified graphene (CMG-R) was synthesized to reduce the surface hydrophilicity of graphene and increase compatibility with the PP matrix. Fourier transform-infrared spectroscopy and scanning electron microscopy were performed to analyze the nanofillers. X-ray diffraction was performed to determine the interlayer spacing of the nanofiller resulting from surface treatment. Differential scanning calorimetry was used to analyze the crystallinity of the nanocomposites. The results indicated that the improved hydrophobicity of the nanofiller due to alkylation reduced the maximum moisture absorption of the PP nanocomposites by 15% compared to PP composites. The findings of this study are useful for reducing fogging in automotive headlamps.

## 1. Introduction

Nanofiller-reinforced polymer nanocomposites have been studied for use in various fields, including automotive, architecture, and electronics. Examples of nanofillers for reinforcement include graphene, carbon nanotubes, whiskers, nano-talc, mica, and montmorillonite (MMT) [1,2,3,4,5,6]. The mechanical properties of polymer nanocomposites generally depend on the particle size, degree of dispersion, interfacial characteristics, and additive content [7]. Incorporation of nanofiller has a significant effect on the melt flow and viscosity of composite and parts processing [8]. The surface treatment of nanofillers is being actively researched as uniform dispersion and interface characteristics are crucial to achieving superior physical properties compared to general polymer composites [1,2,3,4,5,6,7,8]. Graphene, a nanomaterial made of two-dimensional single layers of pure carbon, has recently emerged as a nanofiller. It has remarkable properties such as excellent electrical conductivity, thermal properties, mechanical strength, moisture barrier properties, and wear resistance. Owing to these advantages, it is employed in various industries [9,10,11,12,13].

Polymer composites have attracted attention because international environmental and fuel efficiency regulations require lightweight automotive systems. Polypropylene (PP) is a commercial polymer that is widely used in several industries owing to its low cost and light weight [14]. In particular, PP–talc composites are used in the internal, external, and electric components of automotives, owing to their low cost and excellent formability. Headlamps that assist in safe night driving have also evolved because of recent advancements in automotive technology. For more than a century, headlamps have served to illuminate highways under low-light conditions. Although headlamps have recently transformed beyond standard safety devices, their primary role of ensuring a proper field-of-view during night driving has not changed. The internal temperature of headlamps increases via the operation of the light source, and condensation occurs because of the temperature difference with the external environment. This in turn occurs because of the moisture inside the headlamp’s components [15,16,17], thereby requiring strategies to mitigate moisture absorption of such components. The moisture resistance of polymers can be improved by mixing an inorganic lamellar filler with an aspect ratio sufficient to alter the diffusion path of water molecules [4,5,6,18,19].

PP–talc composites are used in the headlamps’ housing components and are thought to be the primary source of moisture in headlamps. An organically modified graphene filler is manufactured in this study to generate PP nanocomposites with poor hygroscopic properties. An alkyl group with high compatibility with PP is grafted to enhance the dispersibility of the graphene nanofiller [20]. The characteristics of the nanofiller created are then analyzed, and the moisture absorption and thermal characteristics of the PP nanocomposites are investigated

## 2. Materials and Methods

### 2.1. Materials

Octan-1-amine (>98.0%), decan-1-amine (>98.0%), and octadecan-1-amine (>98.0%) were obtained from TCI (Tokyo, Japan) and used without additional pretreatment. PP was prepared by mixing J370 (Lotte Chemical Co., Seoul, Republic of Korea) and CB5108 (Korea Petrochemical Ind. Co., Seoul, Republic of Korea) in a 1:1 weight ratio. A KCNAP400 grade talc (KOCH Co., Ltd., Seongnam-Si, Republic of Korea) was used. Chemically modified graphene (CMG) was produced using 150 µm-grade graphite flakes purchased from the Graphene Supermarket (Ronkonkoma, NY, USA).

### 2.2. Preparation of Alkylated CMG (CMG-R)

Graphene oxide was produced using the method presented in [13,21] (described below), after which graphene functionalized with a linear alkyl group (CMG-R) was synthesized through a surface alkylation reaction using three types of alkylamines: octan-1-amine (CMG-R1, eight hydrocarbons per length of a linear alkyl chain), decan-1-amine (CMG-R2, 10 hydrocarbons), and octadecane-1-amine (CMG-R3, 18 hydrocarbons). The designed alkylated CMG (CMG-R) was able to interact with the alkyl chain of PP at the molecular level.

First, 150 μm-grade graphite flakes (30 g, 1 wt equiv.) were added to a mixture of H_2_SO_4_/H_3_PO_4_ (3600:400 mL) and KMnO_4_ (180 g, 3 wt equiv.) at a ratio of 9:1 and stirred at 50 °C for 12 h. When the reaction was completed, the mixture was cooled to room temperature (23 °C) and diluted using ice water (4000 mL) containing 30% H_2_O_2_ (3 mL). Then, the nonoxidized graphite was removed through filtration, followed by centrifugation at 4000 rpm for 2 h to precipitate oxidized graphite. The precipitate was washed at least three times in a mixed solution of DI water (2000 mL), 30% HCl (2000 mL), and ethanol (2000 mL) to obtain an aqueous graphite oxide solution. Finally, the 2 g/L aqueous graphite oxide solution was subjected to ultrasonic treatment using an ultrasonic homogenizer for more than 6 h to obtain a graphene oxide (GO) aqueous solution [13].

Second, to obtain linearly alkylated graphene to enhance the compatibility between graphene and PP, a graphene surface alkylation reaction was performed using the three aforementioned types of alkylamines. Initially, 500 mL of a 0.1% GO aqueous solution was prepared via ultrasonic treatment for at least 2 h using an ultrasonic homogenizer and 10 g of octadecane-1-amine was added to 500 mL of ethanol. Subsequently, the ethanol solution, which had been stirred for 1 h at 60 °C, was mixed with the GO aqueous solution and stirred again for 40 h at 90 °C to initiate a surface alkylation reaction. The reaction product was cooled to room temperature, after which the precipitate was obtained via centrifugation, washed five to eight times with ethanol, and filtered to obtain graphene. The precipitate was dried in a convection oven at 60 °C for 4 h to obtain functionalized graphene (CMG-R3) with a linearly alkylated surface and affinity toward PP in terms of the molecular structure. CMG-R1 and CMG-R2 were also obtained after performing surface functionalization reactions by varying the alkylamine type using the same method.

### 2.3. Preparation of Nanocomposites

The PP/CMG-R nanocomposites were fabricated using twin-screw extruders (L/D = 40/1, 32 mm, Unee Plus Co., Hwaseong-si, Republic of Korea) with an extruder temperature set to 210 °C at the feeder and 200 °C at the die hole with a rotor speed of 400 rpm. First, PP was supplied to the main feeder at 30 kg h^−1^ and additionally supplied to the side feeder to achieve a talc content of 25 wt% (base sample). The nanocomposite was then prepared by supplying PP to the side feeder to achieve CMG-R contents of 0.05, 0.1, 0.3, and 0.5 wt%. Samples with thicknesses of 1 mm were prepared for the moisture absorption test via injection molding (NE80 injection machine, Woojin Plaimm Co., Chungbuk, Republic of Korea).

### 2.4. Characterization and Instruments

The nanofillers were analyzed using Fourier transform-infrared (FT-IR; Spectrum Two, PerkinElmer, Waltham, MA, USA) spectroscopy and scanning electron microscopy (SEM; Helios 5 Hydra CX, Thermo Fisher Scientific, Waltham, MA, USA). The interlayer spacing of the nanofiller resulting from surface treatment was determined by X-ray diffraction (XRD; EMPYREAN, Malvern Panalytical B.V, Malvern, UK). XRD using a Cu Kα radiation (λ = 0.1542 nm) source was operated at 40 kV and 30 mA, and the data were selected within the scattering angle ranges (2θ) of 15–40° at a rate of 2°/min. The water contact angle measurements were analyzed with PHEONIX-MT(T) (Surface & Electro-Optics Co., Suwon-si, Republic of Korea) under ambient conditions (23 °C). Differential scanning calorimetry (DSC) was used to analyze nanocomposite crystallinity at a heating rate of 5 °C/min in the range of 50–200 °C. The moisture absorption of the nanocomposites was calculated by measuring their weight before and after absorption experiments, which were conducted by leaving the samples in a chamber (TH-G-180, Jeio Tech, Daejeon-si, Republic of Korea) at 85% RH for ca. 1500 h at different temperatures of 35, 50, and 80 °C. Moisture absorption was determined by taking 15 measurements for each composition and averaging the results.

## 3. Results

### 3.1. Characterization of CMG-R

**Figure 1:**
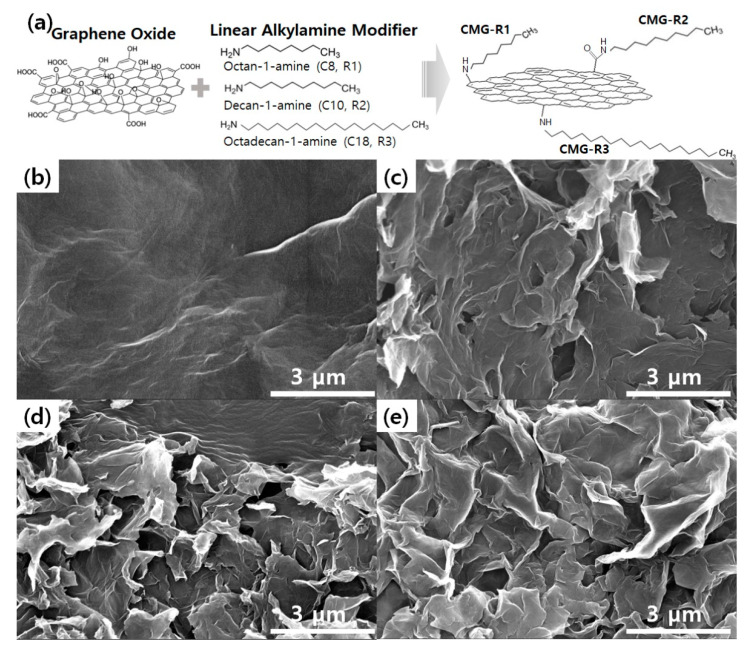
(**a**) Schematic of the graphene surface treatment process. Scanning electron microscopy images of graphene oxide (**b**), CMG-R1 (**c**), CMG-R2 (**d**), and CMG-R3 (**e**).

Figure 1a shows a schematic depicting the reactions of the linear alkylamines (R1, R2, and R3) of various lengths with the graphene oxide surface. The hydroxyl and carboxyl groups of the graphene oxide surface can introduce alkyl groups to the graphene surface by reacting with the amine group of the linear alkylamine. Figure 1b–e shows SEM images of the CMG-R samples according to the length of the alkyl chain. GO (Figure 1b) generally shows a smooth surface, whereas CMG-R forms randomly oriented large aggregated domains. Aggregation is expected to occur when the solvents exhibit more polar dispersibilities than GO, such as ethanol, and this is significantly increased as the alkyl chain length increases [22].

**Figure 2:**
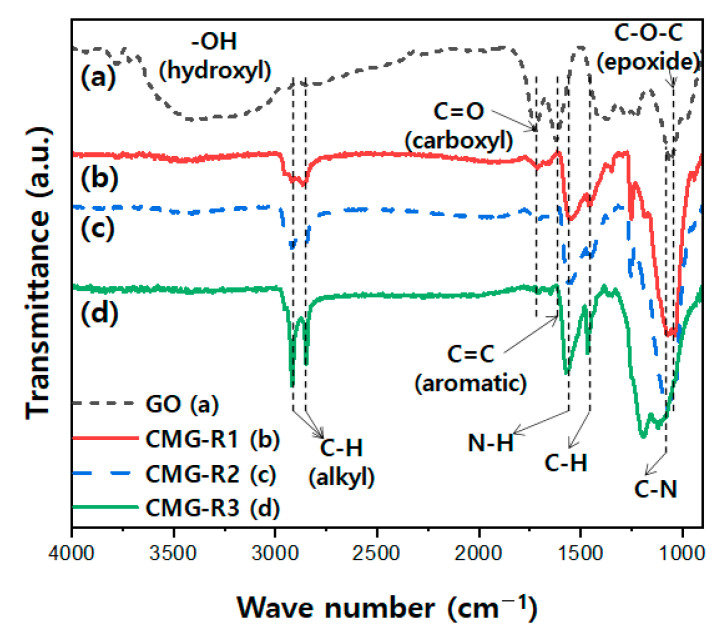
Fourier transform-infrared spectra of graphene oxide (GO) and the CMG-R samples.

Figure 2 presents the FT-IR spectra of GO and CMG-R. As shown in Figure 2 curve (a), GO exhibits peaks at 1725, 1620, and 1055 cm^−1^, corresponding to C=O (carboxyl group), C=C (aromatic group), and C–O–C (epoxide group) [22,23]. As shown in Figure 2 curves (b)–(d), the CMG-R samples have two peaks in the region of 2850–2920 cm^−1^ and one peak at 1486 cm^−1^, which are attributed to –CH of the alkyl group [19,20,21,22,23,24]. It is observed that the intensity of the -CH peak increases with the length of the alkyl chain. Furthermore, peaks denoting C–N and N-H appear at 1080 and 1570 cm^−1^, respectively. However, the peak resulting from the carboxyl group of GO (1725 cm^−1^) is weakened. These FT-IR results demonstrate that alkylation is effectively achieved.

**Figure 3:**
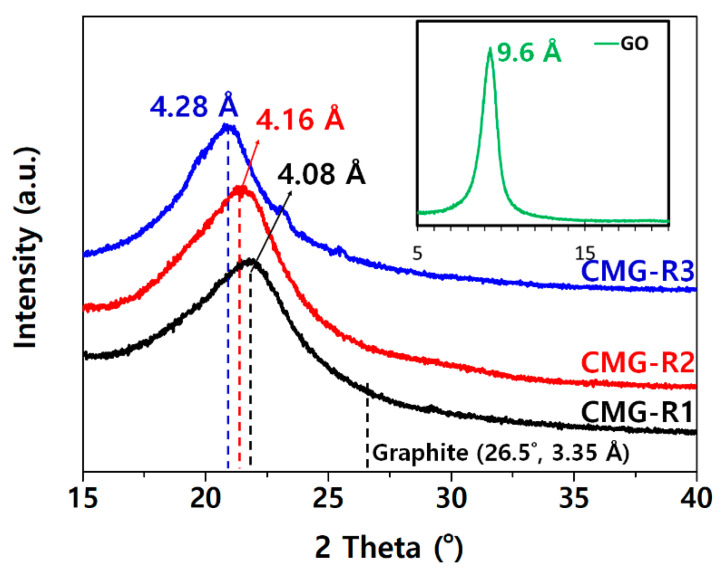
X-ray diffraction patterns of graphene oxide (GO) and the CMG-R samples.

XRD analysis was performed to investigate the interlayer structure of GO and CMG-R, and the distance between graphene was calculated using Bragg’s law [25]:

Bragg’s Law, λ = 2d sinθ (λ = 1.54 Å, Cu Kα)

where λ, d, and θ denote the wavelength of the light source, lattice spacing, and angle between the crystal surface and incident light, respectively. The d-spacing calculated from the 2θ values of the peaks of each sample are 0.960 nm for GO, 0.408 nm for CMG-R1, 0.416 nm for CMG-R2, and 0.428 nm for CMG-R3 (Figure 3). The typical reflection peak (002) of the graphite in graphene oxides, which have their lattice spacing widened because of the oxidation of graphene, is broadened, and the 2θ of the peak shifts to a low angle [1,24,25]. This occurs because oxygen-containing functional groups form on the graphene sheet surface, thereby weakening the van der Waals interactions between the graphene sheets. The peak at (002) is generated after the alkylated reaction has occurred, as the crystal structure of the graphene sheet is restacked by reduction and functionalization. The d-spacing increases as the length of the alkylamine chain increases (Figure 3). This indicates that the graphene sheet does not aggregate as strongly as graphite even after drying, and dispersion is facilitated during compounding with the polymer matrix.

**Figure 4:**
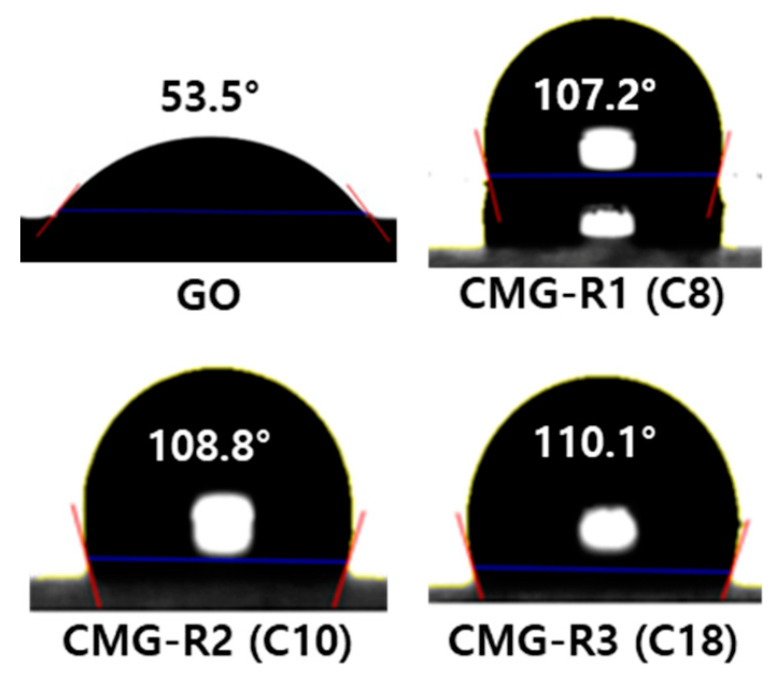
Water contact angle measurements of graphene oxide (GO) and the CMG-R samples.

Figure 4 shows the water contact angle measurements of GO and CMG-R. The surface of GO exhibits hydrophilic properties because of the hydroxyl and carboxyl groups, thereby exhibiting a low contact angle (53.5°). However, the contact angle of the CMG-R surface treated with the linear alkylamine increases owing to the hydrophobicity of the alkyl group. CMG-R3, which has a longer alkyl chain than CMG-R1 and CMG-R2, is expected to be more hydrophobic. Therefore, we selected CMG-R3, which exhibits superior hydrophobic properties, to fabricate the PP nanocomposites.

### 3.2. Characterization of Nanocomposites

**Figure 5:**
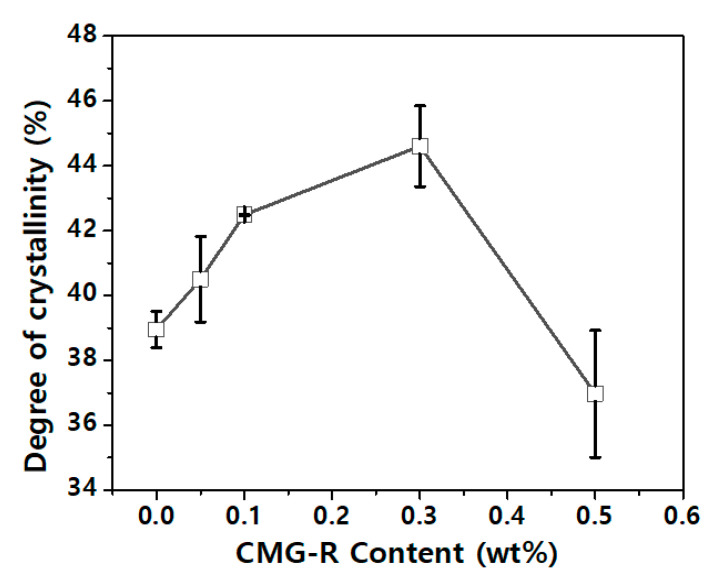
Crystallinity of the PP/CMG-G nanocomposites.

Figure 5 shows the degree of crystallinity of the nanocomposite materials with varying nano-additive contents. The crystallinities of the PP/CMG-R nanocomposites were measured using DSC, which can be calculated using the following equation [26]:

$$X_{c}=\frac{\Delta H_{m}\;\left(m_{t}/m_{p}\right)}{\Delta H_{0}}\times 100$$

where Δ*H_m_* denotes the melting enthalpy, Δ*H*_0_ denotes the theoretical enthalpy of PP at 100% crystallinity (207.1 J g^−1^), *m_t_* denotes the total mass of the sample, and *m_p_* denotes the mass of PP in the sample. As shown in Figure 5, the crystallinity increases with the nano-additive content because a small amount of graphene potentially serves as a nucleating agent to enhance the crystallinity of PP. However, excess graphene (0.5 wt% in this study) has been confirmed to interfere with and reduce the crystallinity of nanocomposites. The crystallinity of the polymer is likely to interfere with moisture diffusion.

**Figure 6:**
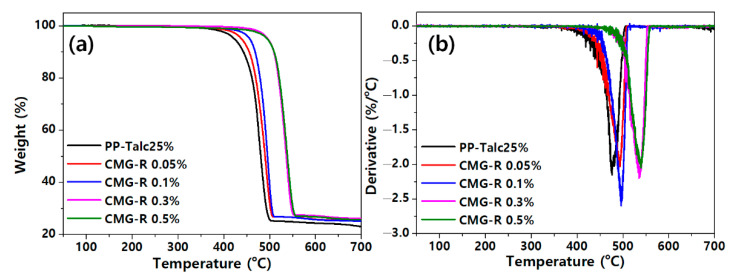
(**a**) Thermogravimetric analysis and (**b**) derivative thermogravimetry curves of the PP/CMG-R nanocomposites.

Figure 6 shows TGA profiles of the PP/CMG-R nanocomposites. The addition of CMG-R enhances the thermal stability when compared to the PP–talc composite. The weight loss that occurs above 420 °C is caused by the gradation of the PP chains and CMG-R decomposition. Notably, clear improvement in the thermal stabilities of the PP nanocomposites is observed with the addition of a small amount of CMG-R. However, thermal degradation occurs rapidly at 0.5% loading. The exact mechanism of the above-mentioned phenomenon has not been elucidated; nevertheless, the thermal stabilities of nanocomposites is improving because the degradation products have difficulty penetrating graphene layers. Therefore, the thermal stabilities of the nanocomposite materials were improved at low filler contents. However, graphene promotes the thermal decomposition of the nanocomposite materials, acting as a heat source domain. Consequently, the thermal stabilities of the PP nanocomposites were significantly enhanced at low CMG-R loadings rather than at high CMG-R loadings [20].

**Figure 7:**
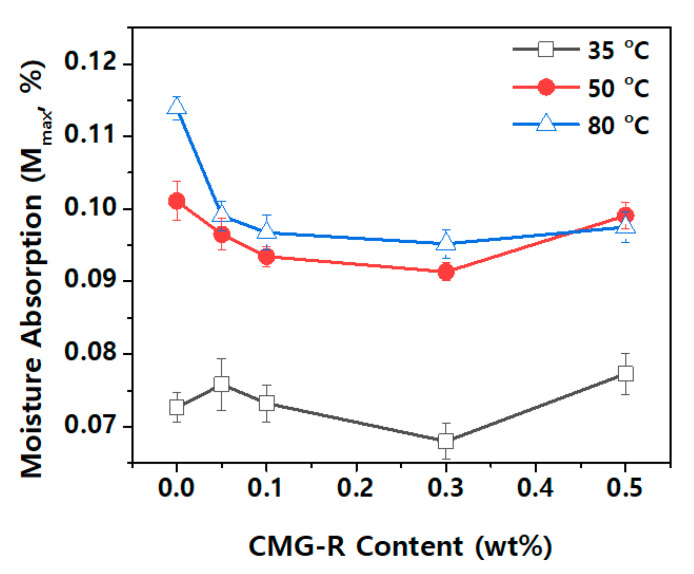
Maximum moisture absorption of nanocomposites with varied CMG-R contents and atmospheric temperatures.

Figure 7 shows the maximum moisture absorption (M_max._) of the nanocomposites based on the CMG-R content and atmospheric temperature. Many studies have been conducted on the moisture absorption behavior of polymer materials [5,19,27,28,29]. Fick’s 2nd Law, a diffusion equation, can be derived from Fick’s 1st Law and the Law of Conservation of Mass. This law explains the diffusion coefficient (D) via the accumulation of water in a sample as a function of time. The diffusion coefficient is fundamentally interpreted as an energy activation process and can be expressed as an Arrhenius relationship that exponentially depends on the activation energy and temperature. Accordingly, as shown in Figure 7, moisture absorption increases as the atmospheric temperature increases.

A lower CMG-R content results in higher moisture absorption (Figure 7) because of the strong hydrophobicity of CMG-R; it prevents moisture dispersion inside the nanocomposite. Furthermore, the moisture in the nanocomposite may be distributed in the overall noncrystalline matrix, including the noncrystalline sections of the polymer and functional groups in CMG-R [5]. Therefore, the increased moisture absorption in CMG-R 0.5 wt% may be attributed to a decrease in the crystallinity of the nanocomposite.

This study verified the moisture absorption reduction effect of the fabricated CMG-R in PP nanocomposites. However, housing fabrication and headlamp assembly are required to measure the effect of decreased fogging in real headlamps. As part of our future work, we intend to fabricate a headlamp with the PP nanocomposites and test its efficacy.

## 4. Conclusions

In this study, graphene-based nanofillers were developed to reduce the moisture absorption of PP composites when applied as housing for automotive headlamps. Reactions with linear alkylamines were used to endow the graphene surface with hydrophobic properties, which were nearly doubled using this process. The moisture absorption in the nanocomposites tended to increase with the surrounding temperature. Furthermore, the moisture absorption decreased with increasing CMG-R content. The effect was caused by the combination of an increase in the crystallinities of the nanocomposite materials with the addition of CMG-R and the reduced hydrophilicity of the additive. As a result, an excess amount (0.5 wt% in this study) of the additive that reduces the crystallinity of the nanocomposite increased moisture absorption. We plan to study the effects of the hydrophobicity and crystallinity of nano additives on moisture absorption through future studies. The PP nanocomposites produced have the potential to reduce fogging in automotive headlamps.

## Data Availability

The data presented in this study are available on request from the corresponding author.
